# Bibliometric and visualization analysis of kidney repair associated with acute kidney injury from 2002 to 2022

**DOI:** 10.3389/fphar.2023.1101036

**Published:** 2023-04-20

**Authors:** Jun Li, Xuezhong Gong

**Affiliations:** Department of Nephrology, Shanghai Municipal Hospital of Traditional Chinese Medicine, Shanghai University of Traditional Chinese Medicine, Shanghai, China

**Keywords:** bibliometrics, acute kidney injury, kidney repair, research hotspots, visualization

## Abstract

**Background:** Renal repair is closely related to the prognosis of acute kidney injury (AKI) and has attracted increasing attention in the research field. However, there is a lack of a comprehensive bibliometric analysis in this research area. This study aims at exploring the current status and hotspots of renal repair research in AKI from the perspective of bibliometrics.

**Methods:** Studies published between 2002 and 2022 related to kidney repair after AKI were collected from Web of Science core collection (WoSCC) database. Bibliometric measurement and knowledge graph analysis to predict the latest research trends in the field were performed using bibliometrics software CiteSpace and VOSviewer.

**Results:** The number of documents related to kidney repair after AKI has steadily increased over 20 years. The United States and China contribute more than 60% of documents and are the main drivers of research in this field. Harvard University is the most active academic institution that contributes the most documents. Humphreys BD and Bonventre JV are the most prolific authors and co-cited authors in the field. The American Journal of Physiology-Renal Physiology and Journal of the American Society of Nephrology are the most popular journals in the field with the greatest number of documents. “exosome”, “macrophage polarization”, “fibroblast”, and” aki-ckd transition” are high-frequency keywords in this field in recent years. Extracellular vesicles (including exosomes), macrophage polarization, cell cycle arrest, hippo pathway, and sox9 are current research hotspots and potential targets in this field.

**Conclusion:** This is the first comprehensive bibliometric study on the knowledge structure and development trend of AKI-related renal repair research in recent years. The results of the study comprehensively summarize and identify research frontiers in AKI-related renal repair.

## 1 Introduction

Acute kidney injury (AKI) is a critical illness characterized by a sudden deterioration of kidney function, initial mortality, high morbidity, and high burden on the health system (Bonventre and Yang, 2011; Susantitaphong et al., 2013). Research on the chronic sequelae of AKI has increased dramatically in the last decade. Recent clinical data suggest that AKI is an important risk influence for progression to chronic kidney disease (CKD) (Coca et al., 2012). Due to the complexity of the structure of the kidney and the incomplete understanding of the pathophysiology of AKI, regenerative medicine for kidney disease is unfeasible. Recovery in surviving patients with AKI depends primarily on the reversal of hemodynamic damage, removal of the cause of kidney injury, and successful repair of the renal parenchyma (Basile et al., 2016). Therefore, developing effective treatment strategies to promote the repair of damaged renal tissue is critical for improving the short- and long-term prognosis of AKI. In the past few decades, much research has revolved around the endogenous repair and regeneration mechanisms of the kidney, including stem cell therapy and exosomes (Fais et al., 2016; Squillaro et al., 2016). In addition, the cellular and molecular mechanisms behind maladaptive repair and renal fibrosis, including epithelial-mesenchymal transformation (EMT), cellular senescence, G2/M cell cycle arrest, fibroblast, and immune cell activation, are constantly updated (Rayego-Mateos et al., 2022). As a result, kidney repair is becoming an important area of AKI research.

At present, bibliometric analysis is mainly used to analyze the development trend, academic exchange and core influence of literature in specific scientific fields (Chen and Song, 2019). Moreover, bibliometric analysis can also be used to explore leading journals in the field, core author teams, and current research frontiers and trends (Merigó and Núñez, 2016; Wu et al., 2021). However, to our knowledge, there is no systematic evaluation exploring kidney repair in AKI. In the present study, we conducted a bibliometric analysis of research related to kidney repair in AKI and explored emerging trends in this field.

## 2 Materials and methods

### 2.1 Data source and search strategy

Web of Science is one of the most authoritative and comprehensive database platforms in the world, with comprehensive content and high quality of academic journals, and is currently the most usually used database for bibliometric analysis (Luo et al., 2022; Zhou et al., 2022). Therefore, Web of Science core collection (WoSCC) was used as the data basis for our article. At the same time, so as to ensure the comprehensiveness and accuracy of the retrieved data, the citation index is selected as SCI-Expanded. The search keyword was “((((TS=(acute kidney injury)) OR TS=(acute kidney failure)) OR TS=(acute renal failure)) OR TS=(acute renal injury)) AND TS=(kidney repair) AND Document types = (ARTICLE OR REVIEW) AND Language = (English)”, and the time span was selected from 1 January 2002 to 31 December 2022. In addition, all valid bibliographic data, including year of publication, title, author name, nationality, affiliation, abstract, keywords, journal name, *etc.*, are saved in plain text format file format in the WoSCC database.

### 2.2 Bibliometric analysis and visualization

Bibliometrics is an independent discipline and provides quantitative methods for reviewing and investigating existing literature in a particular field (Gureyev and Mazov, 2022). During the analysis, detailed information such as authors, keywords, journals, countries, institutions, references, *etc.*, Can be obtained. Visualization helps to uncover the intrinsic connections between this information, such as different authors having the same research topic, research priorities from different institutions, new theories from existing institutions, and so on. For data analysis and visualization, such as country, region co-occurrence, journal dual-maps, high-frequency keyword tendency, co-cited references, and reference bursts, networks between countries, institutional researchers, and co-occurrence analysis, etc., we used both VOSviewer and CiteSpace (Synnestvedt et al., 2005; van Eck and Waltman, 2010). In addition, in the analysis, Taiwan is classified as the People’s Republic of China; England, Scotland, Northern Ireland and Wales are classified as the United Kingdom.

## 3 Results

### 3.1 Trend of global publications and citations

**FIGURE 1:**
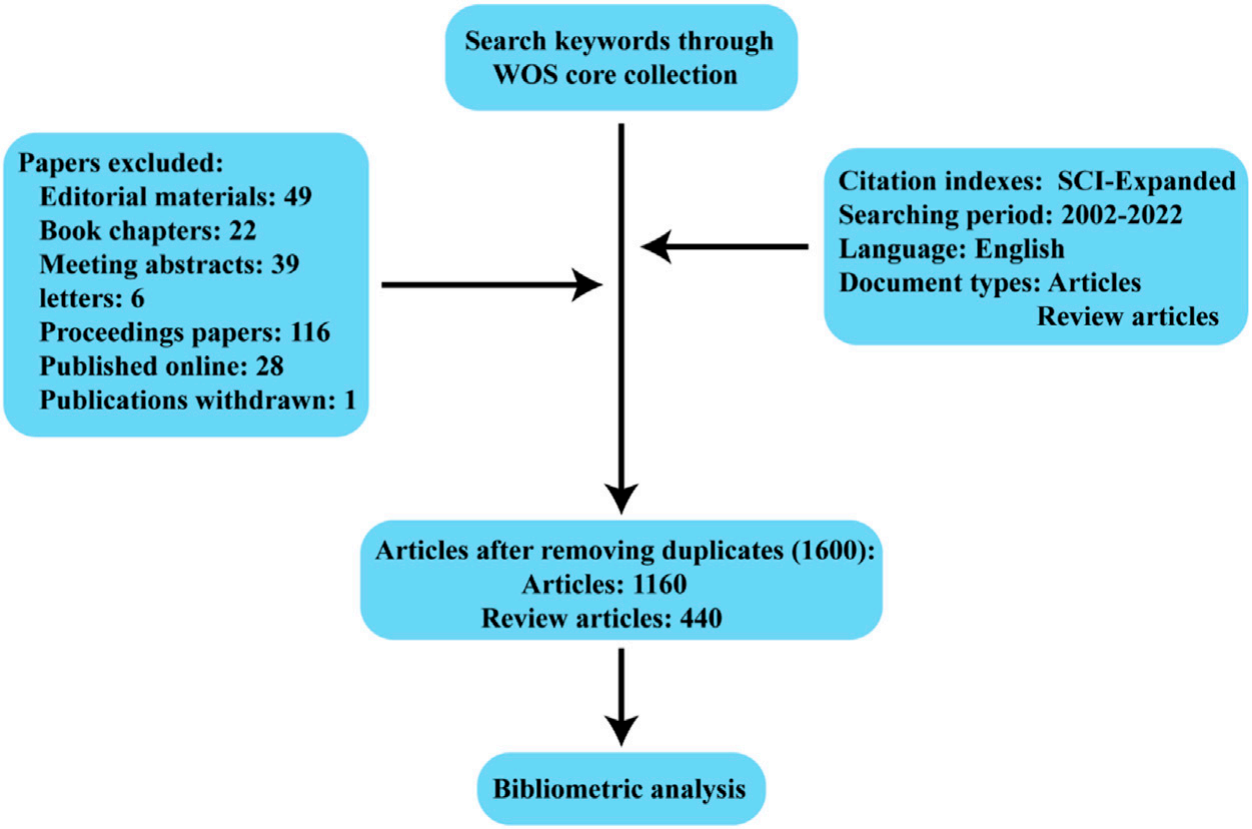
Schematic diagram of the search process.

**FIGURE 2:**
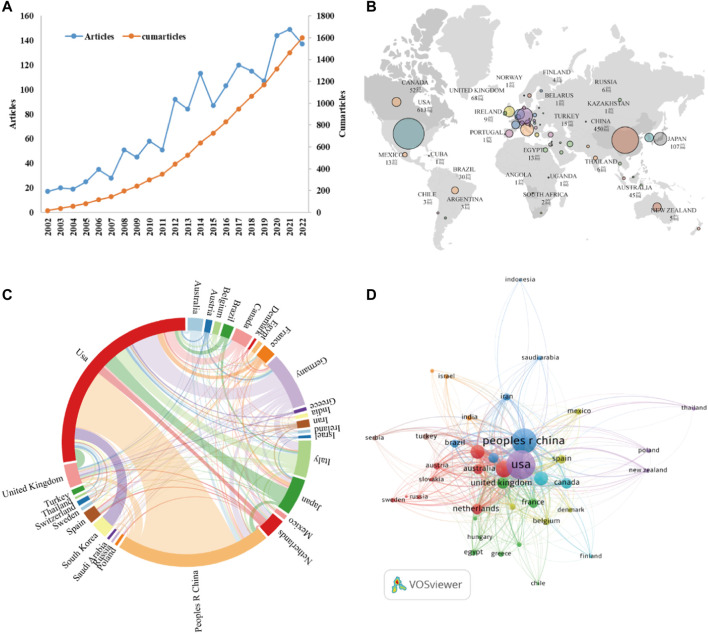
**(A)** The annual number and cumulative publications related to this topic. **(B)** An overview of the number of articles published by country in the world. **(C)** Academic cooperation between countries/regions on this topic. The thickness of segment indicated the frequency of cooperation. **(D)** The citation network of countries/regions mapped through VOSviewer.

Between 2002 and 2022, 1,600 articles (Figure 1) were collected from WoSCC, including 1,160 articles and 440 reviews, and all publications were published in English. The 1,160 papers used in this study were written by 7,893 authors from 1,612 institutions in 56 countries, published in 481 journals, and cited 56,264 reference form 4,528 journals. Over the past 20 years, research on kidney repair in AKI has continuously increased (Figure 2A). These papers were cited an average of 36.57 times per paper, for a total of 73,190 citations.

### 3.2 Countries/regions and institutions analysis

As stated by the world map (Figure 2B), the publications on this topic came from researchers in 56 countries. The specific number of posts and citations of each country is shown in Table 1, among which the United States (613), China (450), Germany (162), Italy (108), and Japan (107) contributed the most. In addition, Italy, the United States, and Germany have higher citation rates than China and Japan. An interactive collaboration map depicts partnerships between countries (Figure 2C). According to Figure 2D, citation relationships between the 30 countries/regions which published no less than nine publications were revealed. Despite publishing more documents, China does not cooperate with other countries as commonly as the United States and Germany.

**TABLE 1:** Top 10 productive countries/regions related to AKI in kidney repair.

Rank	Countries/regions	Documents (N)	Percentage (N/1,600)	Citations	Citations per paper
1	United States	613	38.43	37,333	60.90
2	China	450	28.12	14,058	31.24
3	Germany	162	10.12	11,187	69.06
4	Italy	108	6.75	9,291	86.02
5	Japan	107	6.68	3,391	31.69
6	United Kingdom	68	4.25	3,829	56.31
7	Netherlands	59	3.68	3,165	53.64
8	Canada	52	3.25	1716	33.00
9	South Korea	48	3.00	1979	41.23
10	Australia	45	2.81	2,166	48.13

**FIGURE 3:**
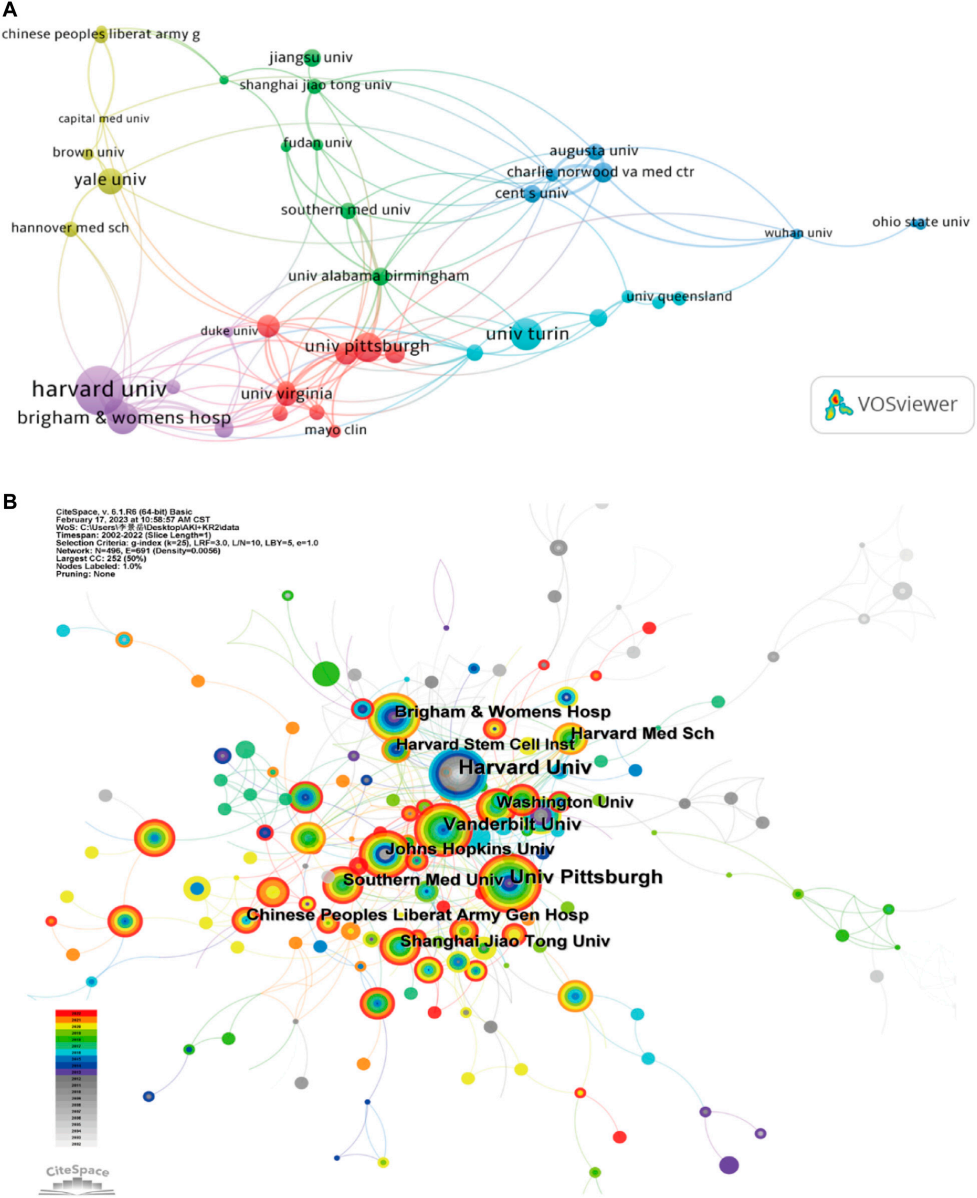
**(A)** Institutional cooperation diagram based on VOSviewer. **(B)** Institutional citation network generated by CiteSpace.

On the word of CiteSpace, 1,600 papers were provided by 1,612 different institutions. Table 2 lists the top 10 institutions with the most articles. It can be found that these 10 institutions are all from the United States and China, which is also in line with the country’s ranking of the number of documents. In institutional cooperation map with VOSviewer (Figure 3A), there is closer cooperation between various U.S. institutions, centered on Harvard University, than Chinese institutions. In terms of citation analysis of institutions, the top three institutions with the most citations are Harvard University (8,885), Brigham and womens hospital (3,424), and Univ Pittsburgh (2,801) (Figure 3B). In addition, the above chart shows that Harvard’s work in this area of research started earlier, while the impact of the research from Brigham and womens hospital was stronger.

**TABLE 2:** Top 10 institutions ranked by the numbers of publications.

Rank	Institutions	Documents (N)	Citations	TLS	Countries/regions
1	Harvard Univ	51	8,885	42	United States
2	Univ Pittsburgh	50	2,801	37	United States
3	Vanderbilt Univ	33	1,692	24	United States
4	Yale Univ	30	2,207	10	United States
5	Southern Med Univ	28	850	18	China
6	Johns Hopkins Univ	27	1716	15	United States
7	Shanghai jiao tong Univ	27	780	13	China
8	Brigham and womens hosp	26	3,424	44	United States
9	Harvard med sch	26	1,167	30	United States
10	Chinese peoples liberat army gen hosp	26	616	17	China

### 3.3 Authors and co-cited authors

**FIGURE 4:**
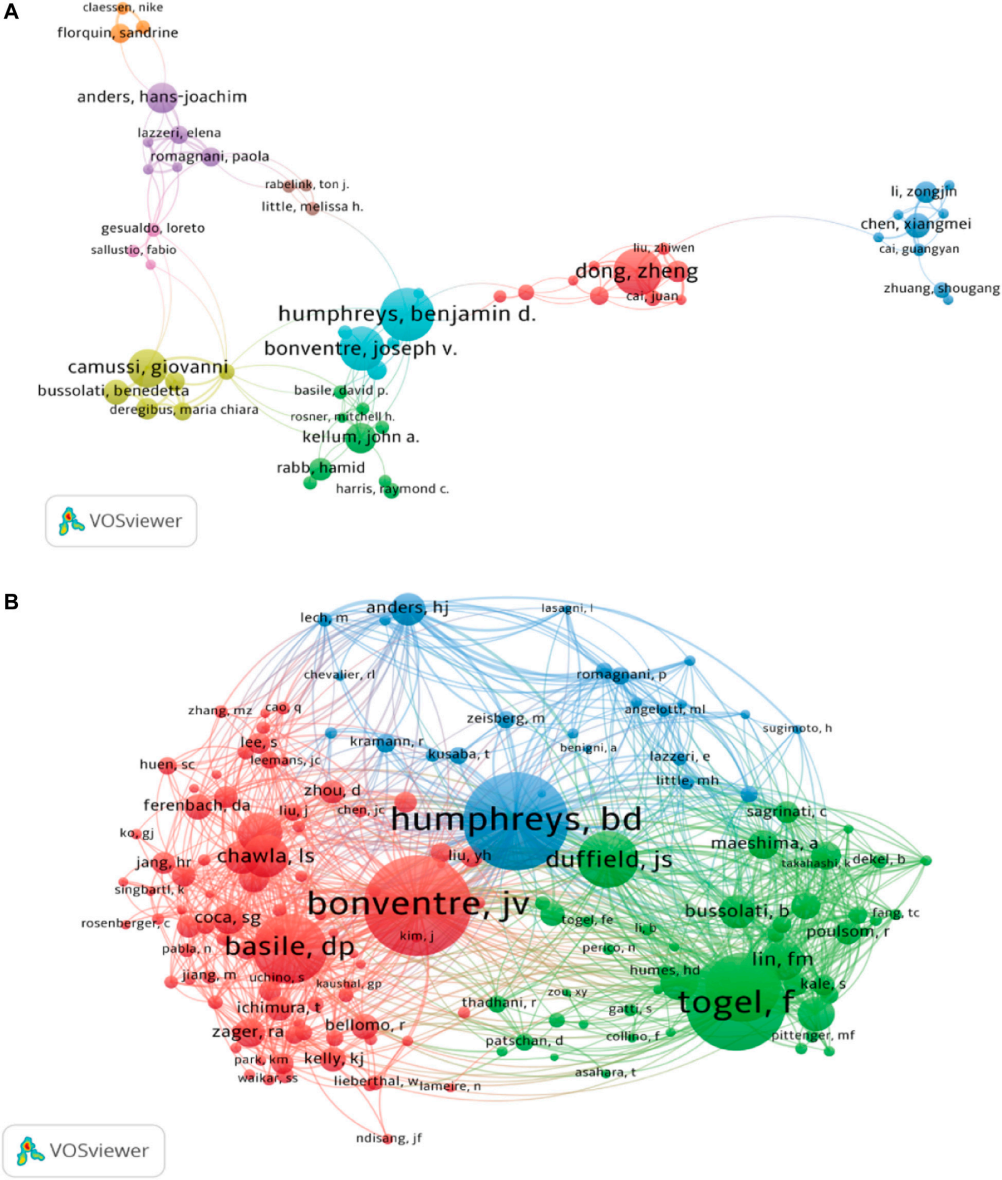
The author cooperation map **(A)** and author co-citation map **(B)** generated by VOSviewer.

A total of 7,893 authors participated in the study of kidney repair at AKI. The top 10 most productive authors and co-cited authors were provided in Table 3. Humphreys BD, Dong Z and Bonventre JV were the top three contributing authors with 30, 27 and 25 publications, respectively. As shown in Figure 4B, the 50 authors with the highest total link strength (TLS) were divided into three main clusters through co-citation analysis. The TLS of Bonventre JV, Humphreys BD and Togel F were 16,837, 18,201, and 17,045, respectively.

**TABLE 3:** Top 10 authors and co-cited authors in the field of AKI in kidney repair.

Rank	Author	Documents	Citations	TLS	Co-cited author	Citations	TLS
1	Humphreys BD	30	2,843	37	Bonventre JV	604	16,837
2	Dong Z	27	1,572	51	Humphreys BD	596	18,201
3	Bonventre JV	25	5,489	20	Tögel F	567	17,045
4	Camussi G	22	3,386	60	Morigi M	418	11,512
5	Kellum JA	17	1,157	19	Baslle DP	416	15,475
6	Anders HJ	17	772	13	Duffield JS	340	10,682
7	Cantley LG	16	1,303	9	Chawla LS	253	7,214
8	Qian H	14	940	55	Yang L	248	7,338
9	Chen XM	14	337	23	Lin FM	234	6,516
10	Bussolati B	14	1,496	21	Herrera MB	209	6,383

### 3.4 Journal analysis

A total of 481 journals published papers on this topic, of which 99 published more than five. In Table 4, the top 10 journals covered 27.63% of the publications, with a total of 442 articles. American Journal of Physiology-Renal Physiology contributed the most articles (n = 97). According to the 2021 Journal Citation Report (JCR), 5 of all top 10 journals are in Q1 in the academic rankings. Two had impact factor (IF) greater than 10, and Kidney International was the journal with maximum IF (18.998).

**TABLE 4:** Top 10 journals associated to the research on kidney repair in AKI.

Rank	Journal title	Documents	Citations	IF	JCR	Country
1	*American Journal of Physiology-Renal Physiology*	97	5,958	4.097	Q2	United States
2	*Journal of the American Society of Nephrology*	82	9,323	14.978	Q1	United States
3	*Kidney International*	50	5,009	18.998	Q1	United States
4	*International Journal of Molecular Sciences*	39	668	6.208	Q1	Switzerland
5	*Scientific Reports*	34	462	4.996	Q2	United Kingdom
6	*PLoS One*	32	1,609	3.752	Q2	United States
7	*Stem Cell Research and Therapy*	30	1,325	8.079	Q1	United Kingdom
8	*Nephrology Dialysis Transplantation*	28	1824	7.186	Q2	United Kingdom
9	*Frontiers in immunology*	25	640	8.786	Q1	Switzerland
10	*Nephron*	25	311	3.457	Q2	Switzerland

**FIGURE 5:**
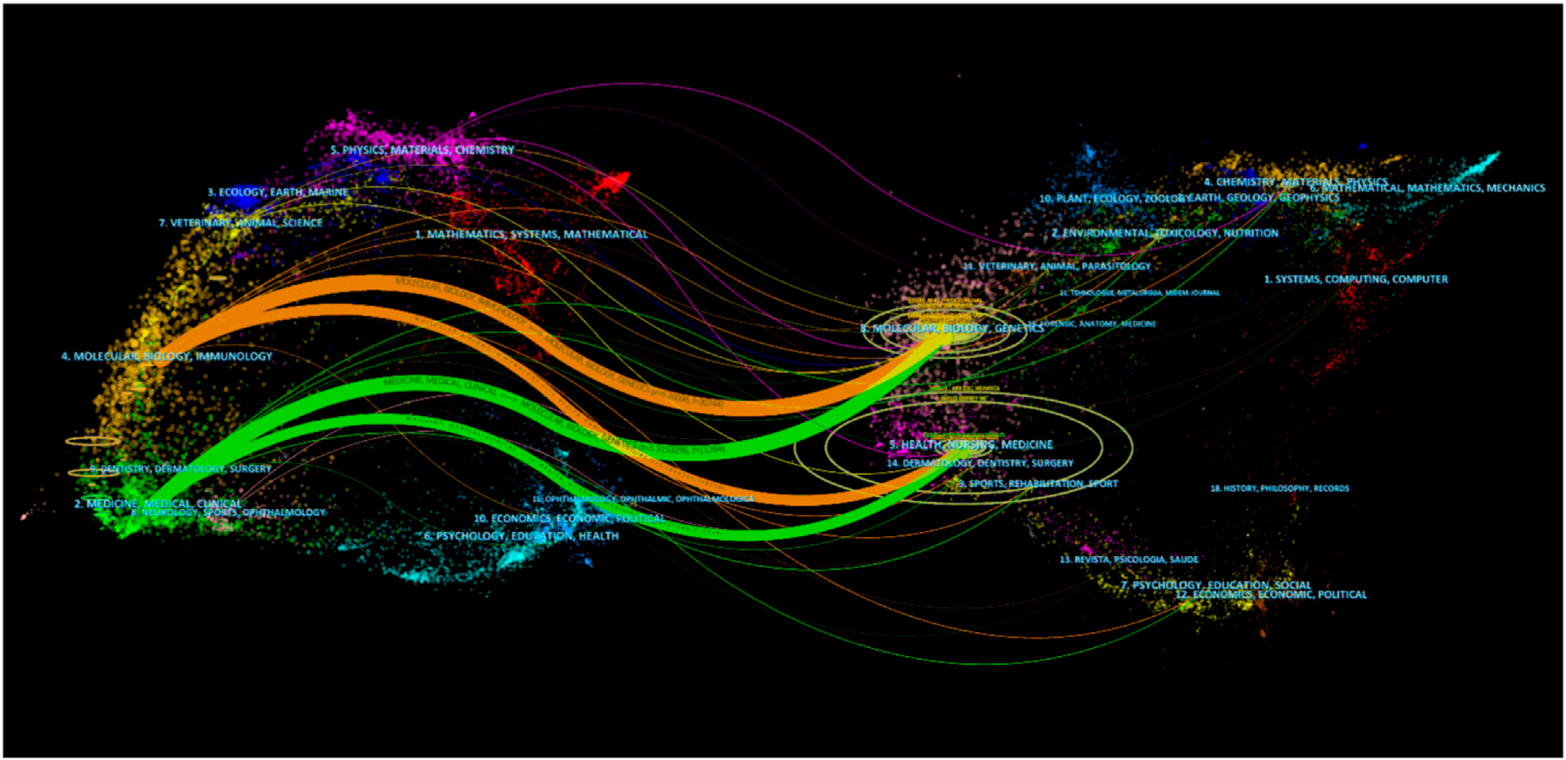
Dual-map overlap of journals on kidney repair in AKI research.

As shown in Figure 5, double-graph overlapping journals were mainly used to visualize the citation path between the cited journal and the cited journal. The citation path started with the cited journal in the left half and ended with the cited journal in the right half, while the topics covered by the journal are marked. It could be found that publications on the themes of “Health, Nursing and Medicine” were most often quoted, establishing two main citation paths that started with “Molecular, Biology and Immunology” and “Medicine, Medical and Clinical”.

### 3.5 Analysis of co-cited references

In total, 1,600 articles under this topic cited 56,264 references. In Table 5, the article entitled “Intrinsic epithelial cells repair the kidney after injury” by Humphreys BD had the most co-citations (n = 233) (Humphreys et al., 2008). Tögel F’ paper “Administered mesenchymal stem cells protect against ischemic acute renal failure through differentiation-independent mechanisms” ranked the second with 215 co-citations (Tögel et al., 2005).

**TABLE 5:** The top 10 co-cited references involved in kidney repair in AKI.

Title	First author	Journal	Year	Citations
Intrinsic epithelial cells repair the kidney after injury	Humphreys BD	Cell Stem Cell	2008	233
Administered mesenchymal stem cells protect against ischemic acute renal failure through differentiation-independent mechanisms	Tögel F	Am J Physiol Renal Physiol	2005	215
Cellular pathophysiology of ischemic acute kidney injury	Bonventre JV	J Clin Invest	2011	197
Mesenchymal stem cells are renotropic, helping to repair the kidney and improve function in acute renal failure	Morigi M	J Am Soc Nephrol	2004	195
Restoration of tubular epithelial cells during repair of the postischemic kidney occurs independently of bone marrow-derived stem cells	Duffield JS	J Clin Invest	2005	181
Epithelial cell cycle arrest in G2/M mediates kidney fibrosis after injury	Yang L	Nat Med	2010	169
Dedifferentiation and proliferation of surviving epithelial cells in acute renal failure	Bonventre JV	J Am Soc Nephrol	2003	131
Stromal cells protect against acute tubular injury via an endocrine effect	Bi B	J Am Soc Nephrol	2007	129
Bone marrow contributes to renal parenchymal turnover and regeneration	Poulsom R	J Pathol	2001	124
Localization of proliferating cell nuclear antigen, vimentin, c-Fos, and clusterin in the postischemic kidney. Evidence for a heterogenous genetic response among nephron segments, and a large pool of mitotically active and dedifferentiated cells	Witzgall R	J Clin Invest	1994	123

**FIGURE 6:**
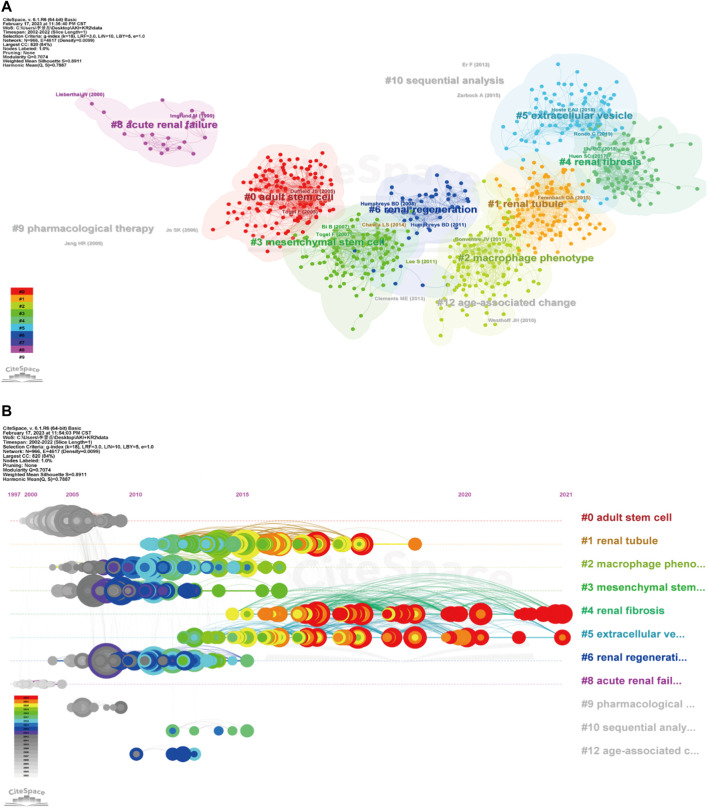
Cluster diagram of co-cited references using keywords as label source **(A)**, and timeline view of co-cited references via title words **(B)** were visualized through CiteSpace.

In Figure 6A, the co-citation network of references was visualized via CiteSpace, and a total of 12 clusters containing keywords were identified. Multiple cell types and cell components are clustered together, including mesenchymal stem cells, renal tubule, extracellular vesicle, macrophage phenotype. In addition, renal fibrosis and age-associated change are also being found in clustering. The timeline view of co-cited references was publicized in Figure 6B, which is important for observing how research hotspots have changed over time. Nine clusters used title words as label source, then displayed different positions and colors on the timeline according to differences in publication time. The recent clusters on the timeline were “#4 renal fibrosis”, “#5 extracellular vesicle”, and “#2 macrophage phenotype”.

**FIGURE 7:**
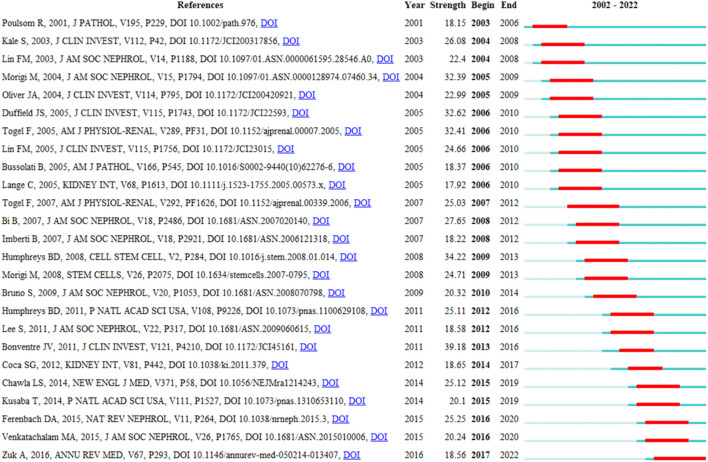
The top 25 references with the strongest citation bursts during 2002–2022.

CiteSpace’s citation burst analysis can uncover studies that have received widespread attention (Synnestvedt, Chen et al., 2005). The 25 references had strongest citation bursts and their most cited time periods were shown in Figure 7. The references that maintain the citation peak until 2020 to 2022 were: “Ferenbach DA, 2015, NAT REV NEPHROL, V11, P264, DOI 10.1038/nrneph.2015.3” (Ferenbach and Bonventre, 2015), “Venkatachalam MA, 2015, J AM SOC NEPHROL, V26, P1765, DOI 10.1681/ASN.2015010006” (Venkatachalam et al., 2015) and “Zuk A, 2016, ANNU REV MED, V67, P293, DOI 10.1146/annurev-med-050214-013407” (Zuk and Bonventre, 2016). Importantly, six of the top 10 co-cited publications are from Prof. Bonventre’s research team, demonstrating the impact of the lab’s research in this field.

### 3.6 Analysis of keywords

**FIGURE 8:**
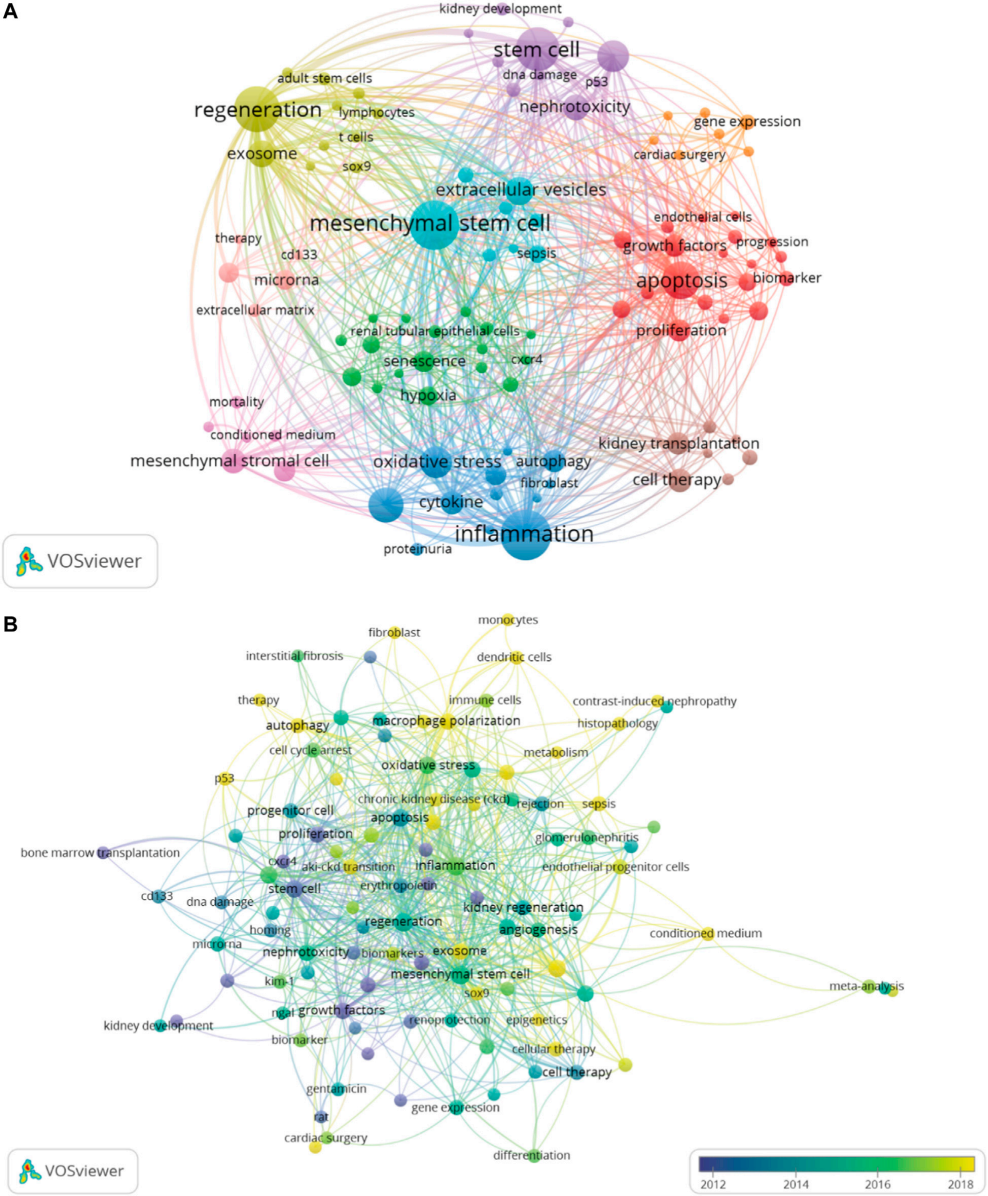
The author keywords co-occurrence network map, which showed the distribution of clusters of major keywords **(A)**. The overlay map displayed that author keywords are colored based on average occurrence time **(B)**. The overlay visualization map showed the color change of the author keywords based on average occurrence time.

The co-occurrence network map and overlay map of the author keywords can explore the cluster distribution characteristics and time variation rules of the keywords. According to Figure 8A, VOSviewer shown the 10 main author keyword clusters. The purple cluster mainly contains several pathological processes related to tubular epithelial cell injury and repair, such as “apoptosis”, “stem cell”, and “mesenchymal stromal cell”. The blue cluster focuses on “Inflammation”, “cytokine”, “oxidative stress and “autophagy”. In the green cluster, cellular senescence is the focus of research attention. In Figure 8B, author keywords appeared in different colors depending on the average year of occurrence. In recent years, “exosome”, “macrophage polarization”, “fibroblast”, “metabolism”, “sox9”, and “aki-ckd transition” appeared frequently, denoting the conversion of AKI to CKD is a current research focus to intervene through multiple pathways.

**FIGURE 9:**
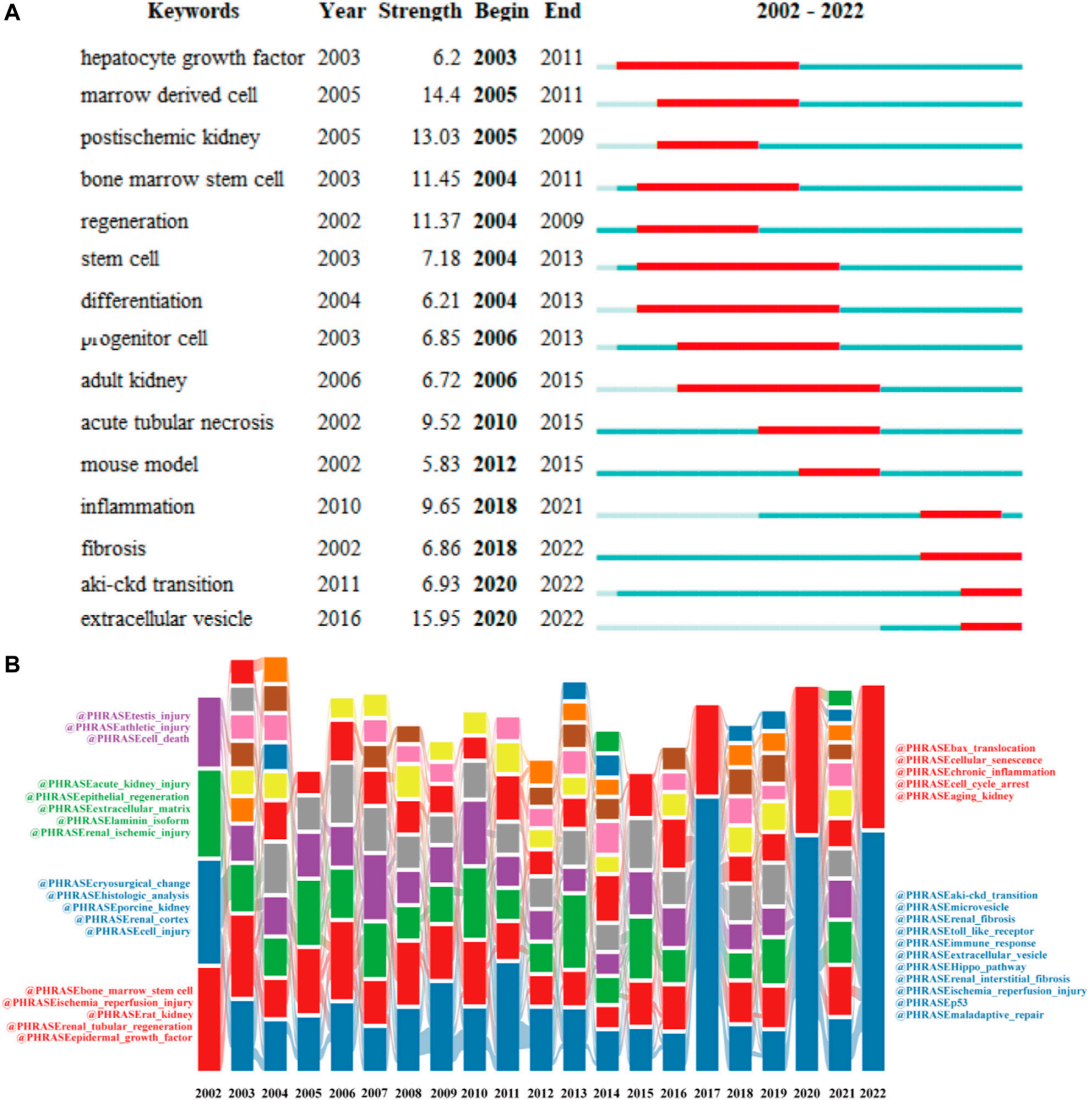
Top 20 keywords with the strong citation bursts **(A)**. Alluvial flow diagrams reflect the flow of concepts to see how concepts change over time **(B)**.

In Figure 9A, we also identified research hotspots by keywords with strong citation outbursts. The citation bursts for keywords like “extracellular vesicles”, “fibrosis”, “aki-ckd transition”, and “inflammation” are still occurring, demonstrating that these words may convert to new hot spots. Alluvial flow diagram can also observe how concepts change over time. In Figure 9B, each of these “blocks” is named after the most critical keyword in the cluster, while the lines connect the same keywords in different years. In addition to showing the flow dynamics of some concepts such as stem cells over time, it can also explore some new research hotspots.

## 4 Discussion

From 2002 to 2022, there has been a rapid increase in the number of annual publications on kidney repair in AKI, indicating that kidney repair will remain a research hotspot in the future. In this study, we provide a comprehensive overview of the development of AKI-related renal repair research through bibliometric analysis, with a focus on possible research hotspots. Globally, the leading countries in kidney repair research are the United States, China, and Germany. This is also supported by an analysis of institutional publications, with 7 of the top 10 productive institutions in the United States and the rest in China (Table 2).

Upon author analysis, Humphreys BD and Bonventre JV were among the top 10 most productive authors and the top 10 most co-cited authors (Table 3), and there is a close collaboration between these two authors (Figure 4A). The most cited study, “Intrinsic epithelial cells repair the kidney after injury,” written by Humphreys BD, Valerius MT, et al., focuses on the main mechanisms of post-ischemic tubular injury in adult mammalian kidneys (Humphreys, Valerius et al., 2008). The work of Humphreys BD’s team also includes interventions in renal fibrosis, cell cycle arrest, and stem cell therapy to facilitate the repair of damaged kidneys after AKI (Liu et al., 2014; Humphreys et al., 2016; Kefaloyianni et al., 2016). The second most cited study, “Administered mesenchymal stem cells protect against ischemic acute renal failure through differentiation-independent mechanism,” written by Tögel F, Hu Z, et al., is on the prevention of ischemic AKI by administering mesenchymal stem cells (Tögel, et al., 2005). The third most cited study, “Cellular pathophysiology of ischemic acute kidney injury,” is a review written by Bonventre JV, Yang L, et al., which elaborates on the cytopathophysiological processes of ischemic AKI and repair (Bonventre and Yang, 2011). Moreover, Bonventre JV’s team studied the molecular markers related to AKI (Han et al., 2002). Additionally, Humphreys BD and Bonventre JV are in the Renal Division, Department of Medicine, Brigham and Women’s Hospital, Harvard Institutes of Medicine. This also shows the dominance of Brigham and Women’s Hospital in this field.

On the basis of the top journals, the *American Journal of Physiology-Renal Physiology*, *Journal of the American Society of Nephrology*, and *Kidney International* had the greatest number of publications on kidney repair in AKI, and these had guidelines for manuscript proposal (Table 4). This study also illustrated that the top 10 journals on the topic included professional journals in the field of nephrology and journals in other fields like stem cell research, and molecular science, indicating that there is a multidisciplinary development trend in this field.

Citation burst, a phenomenon whereby terms such as references and keywords receive a lot of attention and reflect dynamic changes over a certain period of time, was seen. In Figure 7, the 25 references with the strongest citation bursts had a relatively short active period of up to 5 years. The following 3 articles were the most recent of the 25 citation bursts: “Zuk and Bonventre, 2016, ANNU REV MED, V67, P293, DOI 10.1146/annurev-med-050214–013407” describes the complex pathophysiological mechanisms of AKI, biomarkers and therapeutic strategies to prevent the progression of AKI to CKD (Zuk and Bonventre, 2016); “Venkatachalam MA, 2015, J AM SOC NEPHROL, V26, P1765, DOI 10.1681/ASN.2015010006” reviews the progression mechanism of further deterioration of renal structure following AKI transformation to CKD (Venkatachalam, et al., 2015); “Ferenbach and Boventre, 2015, NAT REV NEPHROL, V11, P264, DOI 10.1038/nrneph. 2015.3” explores the archetypal mechanisms by which severely damaged kidneys regenerate and how adaptive repair processes can become maladaptive (Ferenbach and Bonventre, 2015).

Typically, keywords are used in bibliometrics to get an overview of the development of a field. Figure 8B (which colors keywords according to the average year they appear) and Figure 9 show how kidney repair research has evolved over time. The above data shows that the study on kidney repair after AKI can be mainly divided into two important development directions. The first research direction focused on the restoration and regeneration of epithelial cell function. The acute phase of AKI is characterized by cell death, followed by a recovery phase, in which there is an activation of protective and regenerative mechanisms in the surviving cells to restore the properties and functions of epithelial cells (Ruiz-Ortega et al., 2020). A range of cells, such as resident renal progenitor cells and stem cells, are thought to contribute to kidney repair and regeneration (Rayego-Mateos, et al., 2022). Renal injury leads to local microenvironment recruitment and activation of progenitor cells and cell-dependent tissue repair (Huang et al., 2021). Progenitor cells of the proximal tubule are more resistant to death and replace injured cells by differentiating into tubular epithelial cells (Gupta et al., 2006; Lazzeri et al., 2018). In addition to renal progenitor cells, subsequent studies have found that circulating bone marrow stem cells and kidney-resident stem cells also contribute to kidney repair and regeneration (Li et al., 2010). Therefore, the regenerative potential of pluripotent embryonic stem cells (ESCs), induced pluripotent stem cells (iPSCs), and mesenchymal stem cells (MSCs) have been gradually emphasized in AKI research. Recent studies have found that the nephroprotective effects of stem cell transplantation may depend on its paracrine effects, including the release of growth factors, chemokines, cytokines, and extracellular vesicles to induce proximal tubular cell proliferation, dedifferentiation, and angiogenesis (Ranghino et al., 2017; Liu et al., 2020). At present, extracellular vesicles and exosomes are still potential research hotspots. The second research direction focuses on the exploration of renal repair mechanisms dominated by renal fibrosis. After kidney injury, various intrinsic repair processes are rapidly activated, and some pathophysiological processes constitute maladaptive repair, which may promote fibrosis and lead to a progressive decline in renal function (Rayego-Mateos et al., 2021). The major role of oxidative stress in the progression of AKI to CKD initially attracted attention in research. Defense mechanisms against hypoxia and oxidative stress in cells, such as hypoxia-inducible factor (HIF) and nuclear factor E2-related factor 2, are considered likely to be suitable therapeutic targets (Kim et al., 2009; Kapitsinou et al., 2012). Subsequently, other signaling pathways activated due to maladaptive repair like mitochondrial dysfunction and epigenetic changes have been found as potential therapeutic targets for intervention in renal fibrosis after AKI (Cianciolo Cosentino et al., 2013; Stallons et al., 2014). In recent studies, a variety of pathological mechanisms are closely related to maladaptive kidney repair, including macrophage polarization, cell cycle arrest, and cell senescence. In addition, new molecules and pathways, such as the Hippo signaling pathway, Sox9, and p53, have also begun to receive attention in this field. In addition, activation of the senescence mechanism in renal tubular cells and the accumulation of senescent cells will lead to the failure of regeneration after AKI to CKD conversion (Gire and Dulic, 2015; Kim et al., 2019). Overall, CiteSpace’s keyword exploration also helps reveal future trends in space. We concluded that extracellular vesicles (including exosomes), macrophage polarization, and Hippo pathway may be current research hotspots, which will be described in detail below. Additionally, cell cycle arrest, cellular senescence, and Sox9 are also potential research directions and targets.

### 4.1 Extracellular vesicles (EVs)

Besides soluble cytokines, EVs such as exosomes (30–100 nm in diameter) and microvesicles (100–1,000 nm in diameter) have been described as a different form of cell-to-cell communication through the horizontal transfer of microRNAs (miRNAs), mRNAs, and proteins-to-target cells (Lu et al., 2022). Apart from the stem cells mentioned above, all regions of the nephron can release EVs. Since the study by Camussi et al. found that microvesicles from mesenchymal stem cells (MSCs) may activate proliferation in renal tubular cells surviving injury through horizontal transfer of mRNA, there is increasing evidence supporting the involvement of EVs in pathophysiology and repair process of AKI (Bruno et al., 2009; Camussi et al., 2010). Research have found that renal proximal tubular epithelial cells (RPTECs) of injured kidneys can release EVs carrying miR-216a and stimulate nearby RPTECs for epithelial-mesenchymal conversion via the PTEN/Akt pathway, followed by renal interstitial fibrosis (Qu et al., 2019). Tubular cells can also communicate bidirectionally with other cell types involved in kidney injury through EVs. For example, damaged PTECs release EVs, which stimulate macrophage activation and infiltration and amplify the inflammatory response (Lv et al., 2018). Specifically, EVs can influence processes such as inflammation, apoptosis, autophagy, oxidative stress, and cell proliferation, thereby contributing to the development of AKI (Han and Lee, 2019).

Over the past few years, EVs have received a lot of attention as a promising pro-regenerative entity and possible alternative to cell therapy due to their involvement in cell differentiation, proliferation, and angiogenesis, as well as the regulation of extracellular matrix turnover during regeneration. There is considerable evidence that EVs have a nephroprotective effect on improving kidney injury in different experimental AKI models. Various AKI models have shown the protective effects of EVs derived from bone marrow MSCs (BM-MSCs). MiR-199a-3p derived from BM-MSC-EVs prevents renal I/R injury and apoptosis by modulating the Akt and Erk1/2 signaling pathways (Zhu et al., 2019). The important role of miRNAs transferred by BM-MSC-EVs in post-AKI recovery and regeneration was also revealed, while miRNA depletion significantly reduced its intrinsic regeneration potential in AKI (Collino et al., 2015). In different AKI models, umbilical cord MSC (UC-MSC)-derived EVs have also been shown to be beneficial. UC-MSC-derived EVs can enhance proliferation and angiogenesis in a HIF-1α-independent manner to improve renal function after unilateral I/R (Zou et al., 2016). Furthermore, UC-MSC-EV can also promote recovery from kidney injury by increasing HGF expression, and its mechanism may be related to the transfer of RNA to injured renal tubular cells and activation of Erk1/2 signaling to accelerate the dedifferentiation and growth of renal tubular cells (Ju et al., 2015). In addition to the above sources, the effect of EVs derived from human placental MSCs, adipose MSCs, liver MSCs, and macrophages and endothelial progenitor cells have also been studied in post-AKI regeneration and recovery, and some new evidence has been obtained (Gao et al., 2020; Grange et al., 2020; Zhang et al., 2020). Several promising approaches are also being explored, including modifying the EVs of MSCs to enhance their targeting of the kidneys, or artificially altering the active molecules delivered in the EVs. Although the mechanisms of current EVs-based studies remain murky in most cases, many examples of beneficial effects dominated by MSC-derived EVs suggest that they have great potential to be used as a basis for the development of novel post-AKI renal repair and regenerative therapies.

### 4.2 Macrophage polarization

Macrophages are the main immune cells in the normal kidneys and are thought to be core members in the pathogenesis of AKI. Macrophages can change their phenotype according to the surrounding microenvironment. Macrophages that infiltrate the kidneys have a profound effect on kidney damage, repair, and fibrosis due to different polarization states (Lee et al., 2011). Typically, macrophages accumulate in the kidneys after injury and undergo a transition from a pro-inflammatory M1 phenotype to an alternatively activated M2 phenotype (Engel and Chade, 2019). Therefore, M2 macrophage polarization is essential for inflammation inhibition, remodeling, and AKI recovery. Macrophage depletion in the later stages of the Ischemia-reperfusion injury (IRI) model reduces PTECs proliferation and delays renal repair, while IL-4 polarizes metastasis-induced repair of M2 macrophages (Vinuesa et al., 2008; Meng et al., 2022). Interestingly, PTECs can also modulate the phenotype of macrophages. In sepsis-induced AKI, CSF2 secreted by injured TECs can facilitate the transition from M1 to M2 macrophages in a dose- and time-dependent mode (Li et al., 2020). The mechanism by which macrophages promote tubular repair is complex. Macrophage-derived Wnt7b, BRP-39, and IL-22 have been identified as factors that directly promote tubular repair after ischemic injury (Lin et al., 2010; Kulkarni et al., 2014). Vascular-resident CD169+ macrophages limit neutrophil infiltration after ischemic damage by down-regulating the expression of intercellular adhesion molecule-1 on vascular endothelial cells, and they indirectly promote tubular repair (Karasawa et al., 2015). Therefore, macrophage phenotype plays a pivotal role in kidney repair. However, it is difficult to maintain a macrophage phenotype for a successful switch *in vivo*. Lipoclysin-2 (Lcn-2) is an effective regulator of macrophage polarization that stabilizes the M2 macrophage phenotype (Mertens et al., 2020). Additionally, when kidney injury is unresolved and progressive, M2 macrophages can become profibrotic through mesothelial-to-mesenchymal transition (Huen and Cantley, 2015). Overall, targeting the regulation of macrophage phenotypes is a promising treatment to promote renal repair after AKI. Although some details remain to be clarified, such as the precise molecular characteristics and cell fate plasticity of macrophage subsets *in vivo* after AKI, the direct extrapolation of relative M1/M2 expression profiles defined *in vitro* and its biological effects on *in vivo* repair responses remain to be confirmed.

### 4.3 Cell cycle arrest and cellular senescence

The mitotic cell cycle consists of four stages, including G0-G1, S, G2 and M, and is controlled by four checkpoints, including G1/S, S, G2/M and M (Hartwell and Weinert, 1989). However, failure to pass one of these checkpoints can induce cell cycle arrest or cell death at a specific stage. Under physiological conditions, the rate of renal cell turnover is very low, and RPTECs are mostly maintained in the G0-G1 phase (Canaud and Bonventre, 2015). After kidney injury, surviving RPTECs enter the cell cycle and divide to replace the damaged cells. Early research suggests that cell cycle arrest may be a protective mechanism for post-AKI RPTECs, promoting DNA damage repair or preventing cell division when DNA damage cannot be repaired (De Chiara et al., 2021). Studies have found that p21 is induced in the kidneys after several types of AKI, including cisplatin administration, ischemia-reperfusion, and ureteral obstruction, and causes cell cycle arrest. However, the deletion of the p21 gene leads to more severe kidney injury (Megyesi et al., 1998; Megyesi et al., 2001). The Bonventre laboratory study confirms that G2/M cycle arrest of epithelial cells exacerbates the renal fibrotic process in ischemic, toxic and obstructive AKI models; however, JNK or p53 inhibitors can rescue the subprocess (Yang et al., 2010). Additionally, prolonged blockade of the G1 or G2/M stages after AKI leads to senescence-associated secretory phenotype (SASP), which includes the secretion of profibrotic and pro-inflammatory factors, exacerbating kidney damage and forming a vicious cycle (Yu and Bonventre, 2020). Targeting cell cycle arrest is a potential innovative strategy to improve kidney repair and prevent CKD, as studies targeting p21, p53, Cyclin G1 (CG1) and HDAC inhibitors have progressed in animal experiments (Skrypnyk et al., 2016; Fu et al., 2017; Canaud et al., 2019). With the further recognition of the importance of cell cycle abnormalities in renal repair after AKI, therapeutic strategies that target this process deserve the attention of researchers.

### 4.4 Hippo signaling pathway

The Hippo pathway is highly evolutionarily conserved in mammals and consists of a three-step kinase cascade of MST1/2 (also known as STK4/3), LATS1/2, and YAP/TAZ (also known as WWTR1) (Callus et al., 2006). This pathway regulates cell growth and fate decisions, organ size, and regeneration through phosphorylation and inactivation of its downstream effector YAP/TAZ (Ma et al., 2019). In recent years, the association between the Hippo pathway and renal repair and fibrosis after AKI has attracted attention. Studies have found that YAP expression and nuclear distribution are enhanced in renal RPTC in AKI patients and I/R mice, while histological recovery is delayed in YAP/TAZ double-knockout mice (Chen et al., 2018). The EGFR-PI3K-Akt pathway and RacGAP1 can help RTECs recover from acute injury by activating YAP (Zhou et al., 2020). There is considerable evidence that YAP may promote proliferation and redifferentiation of reconstituted epithelium after acute I/R-induced AKI (Xu et al., 2016). However, the function of the Hippo pathway in kidney repair after AKI may be bidirectional. Sustained activation of YAP/TAZ in severe AKI impedes redifferentiation of dedifferentiated tubular cells and promotes renal fibrosis, leading to maladaptive repair and CKD (Xu et al., 2021). Epithelial-mesenchymal transformation (EMT), G2/M cell cycle arrest, and macrophage M2 polarization have been associated to Hippo signaling-mediated renal fibrosis (Seo et al., 2016; Feng et al., 2018; Zheng et al., 2021). In summary, proper regulation of the Hippo signaling pathway, especially YAP intervention in repair, may be an effective prevention target for AKI-CKD conversion after AKI. Although the above evidence is convincing, the correlation between the hippo pathway and AKI needs to be further studied, and the underlying mechanisms and pathways need to be further elucidated.

### 4.5 Sox9

The kidneys can regenerate after injury; however, the intrinsic molecular mechanisms remain unknown. The transcription factor Sox9 is important in organogenesis during mammalian embryonic development. Simultaneously, Sox9 is a transcription factor essential for normal kidney development, and recent studies have highlighted the importance of its expression in the repair process of renal tubular epithelial cells (Kumar et al., 2015; Kang et al., 2016). In the early stages of kidney injury, Sox9 expression is significantly upregulated within damaged tubular epithelial cells, and approximately 40% of Sox9-positive cells proliferate and expand after kidney injury. Despite the recovery of kidney function, Sox9 activation was found to persist on day 28. Furthermore, multiple upstream and downstream molecules or mechanisms are involved in the renal repair process via Sox9 in AKI. Sox9 drives the upregulation of VGF nerve growth factor in multiple AKI models and acts as a stress-responsive protective gene in TECs (Kim et al., 2020). In IRI and folic acid (FA)-induced AKI models, early growth response 1 (EGR1) can increase Sox9 expression in kidney TECs by directly binding to the promoter of the *Sox9* gene, consequently promoting Sox9 cell proliferation by activating the Wnt/β-catenin pathway (Chen et al., 2022). A recent study found that kidney-resident Sox9+ TECs can be activated and secrete various factors to promote tissue repair, of which S100A9 may be a key factor associated with the renal repair pathway (Nie et al., 2023). Although the upregulation of Sox9 was identified as an early event after AKI in these studies, the primary player in kidney repair as tubular epithelial cells or resident tubular progenitor cells remains controversial. In addition to being involved in the intrinsic repair of the kidney, Sox9 plays a role in maladaptive repair and chronic fibrosis in the AKI-CKD transition. SOX9 has been associated with human renal fibrosis and is required for experimentally-induced renal fibrosis in mice (Raza et al., 2021). Both embryonic stem cell-derived extracellular vesicles (ESC-EVs) and human amnion-derived mesenchymal stem cells (hAD-MSCs)-derived exosomes activate Sox9 in TECs to promote physiological repair after AKI and inhibit the progression of fibrotic processes (Zhu et al., 2017; Yu et al., 2021). In conclusion, although Sox9 has been associated with kidney repair after AKI, a further in-depth research is still necessary.

## 5 Limitation

First, not all studies from the databases were included and data were taken from the WoSCC database only. Second, we only selected studies and review articles written in English, articles published in non-English or non-research/review articles were not included in this study, leading to some omissions. Third, data were obtained through bibliometric software based on machine learning, which may lead to bias in bibliometric research and discussion.

## Data Availability

The original contributions presented in the study are included in the article/Supplementary Material, further inquiries can be directed to the corresponding author.
